# BCOR-CCNB3 fusion–positive sarcoma treated with chemotherapy and carbon-ion radiotherapy: a case report of long-term disease control of a cervical spine case with a 12-year follow-up

**DOI:** 10.3389/fonc.2026.1869278

**Published:** 2026-06-10

**Authors:** Naoki Oike, Reiko Imai, Akira Ogose, Takashi Ariizumi, Yudai Murayama, Tomohiro Miyazaki, Kazunaga Kimura, Hajime Umezu, Hiroyuki Kawashima

**Affiliations:** 1 Division of Orthopedic Surgery, Graduate School of Medical and Dental Sciences, Niigata University, Niigata, Japan; 2 Department of Radiology, QST Hospital, Chiba, Japan; 3 Department of Orthopedic Surgery, Uonuma Kikan Hospital, Niigata, Japan; 4 Department of Pathology, Graduate School of Medical and Dental Sciences, Niigata University, Niigata, Japan

**Keywords:** BCL6 corepressor, BCOR-CCNB3, carbon-ion radiotherapy, case report, cervical spine, cyclin B3, Ewing sarcoma, undifferentiated round-cell sarcoma

## Abstract

*BCOR–CCNB3* fusion–positive sarcoma (BCS) is a recently recognized subtype of undifferentiated round-cell sarcoma that occurs predominantly in children and adolescents. Owing to its rarity, an optimal treatment strategy has not been fully established, and management has historically followed treatment protocols for Ewing sarcoma. Although radiotherapy is a potential treatment option, the role of carbon-ion radiotherapy (CIRT) in treating this tumor has not been previously reported. A 14-year-old boy presented with posterior neck pain. Magnetic resonance imaging demonstrated a tumor arising from the posterior elements of the third cervical vertebra. Needle biopsy revealed a round-cell sarcoma without EWS gene rearrangement, and the patient was initially treated for Ewing-like sarcoma. Systemic chemotherapy with vincristine, doxorubicin, and cyclophosphamide alternating with ifosfamide and etoposide was initiated, resulting in tumor shrinkage. Subsequently, the patient underwent CIRT (70.4 Gy in 16 fractions), followed by adjuvant chemotherapy. Magnetic resonance imaging after treatment completion showed no evidence of residual tumor. More than 12 years after treatment, the patient has remained asymptomatic without neurological deficits, tumor recurrence, or distant metastasis, although cervical kyphosis was radiographically observed. Subsequent genetic analysis confirmed the presence of the *BCOR–CCNB3* fusion gene. To the best of our knowledge, this is the first reported case of BCS treated with CIRT. This case demonstrates that CIRT can achieve durable local control and suggests that it may represent a promising treatment option for BCS when surgical resection is challenging.

## 1 Introduction

Tumors morphologically resembling Ewing sarcoma (ES) but lacking *EWS* gene alterations have traditionally been classified as Ewing-like sarcomas. Among them, *BCOR–CCNB3* fusion–positive sarcoma (BCS), first described by Pierron et al. in 2012, is a recently recognized subtype of undifferentiated round-cell sarcoma characterized by a *BCOR–CCNB3* gene fusion resulting from a paracentric inversion on chromosome X (1). According to the 2022 World Health Organization classification of soft tissue and bone tumors, sarcomas with BCOR alterations, including BCS, are distinct entities among undifferentiated small round-cell sarcomas (2).

Because of its rarity, an optimal treatment strategy for BCS has not been fully established. Historically, management has been based on protocols for ES and continues to follow a similar multimodal approach consisting of chemotherapy, surgery, and radiotherapy, with comparable oncological outcomes (3). In this context, radiotherapy, including particle beam therapy, may be a treatment option, particularly for unresectable tumors, similar to ES management (3, 4). Although combination therapy with carbon-ion radiotherapy (CIRT) and chemotherapy for ES has demonstrated favorable local control (5), the use of CIRT for BCS has not been reported. Herein, we report a rare case of BCS arising from the cervical spine in which long-term disease control was achieved after systemic chemotherapy and CIRT.

## 2 Case description

A 14-year-old boy presented with neck pain for 1 month. Magnetic resonance imaging (MRI) revealed a mass in the left posterior neck, and the patient was referred to our hospital for further evaluation. On physical examination, no obvious mass was palpable, and no neurological deficits were observed. Contrast-enhanced MRI revealed a mass arising from the posterior elements of the third cervical vertebra (C3), showing low signal intensity on T1-weighted images and high signal intensity on T2-weighted images with heterogeneous contrast enhancement. Compression of the spinal cord was also observed (**Figures 1A–D**). Axial computed tomography at the C3 level demonstrated focal osteolytic destruction at the base of the spinous process (**Figure 1E**).

**Figure 1:**
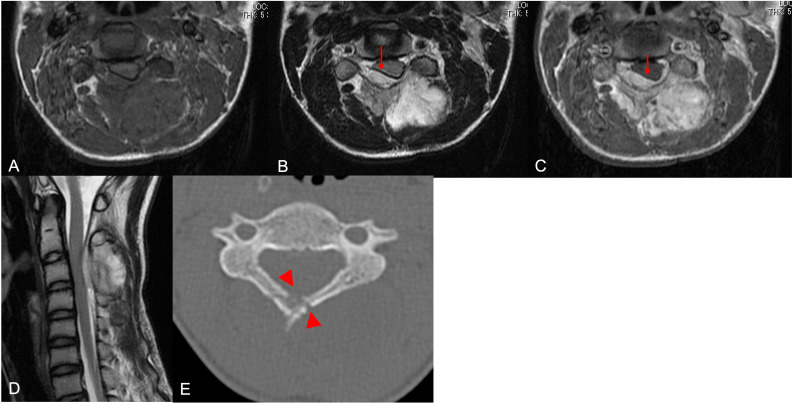
BCOR–CCNB3 fusion-positive sarcoma arising from the posterior elements of the third cervical vertebra (C3). Magnetic resonance imaging (MRI) demonstrates a mass originating from the posterior elements of C3 **(A–D)**, showing low signal intensity on T1-weighted images **(A)** and high signal intensity on T2-weighted images **(B)** with heterogeneous contrast enhancement **(C)**. Spinal cord compression was also observed. Sagittal imaging shows tumor extension from C2 to C4 **(D)**. Computed tomography reveals a focal osteolytic lesion at the base of the spinous process **(E)**.

Needle biopsy revealed an undifferentiated round-cell sarcoma (**Figure 2A**) and CD99 positivity on immunohistochemistry (**Figure 2B**); however, EWS gene rearrangement was not detected. Accordingly, the tumor was classified as an Ewing-like sarcoma. Given the risk of spinal cord compression due to the tumor, systemic chemotherapy with vincristine, doxorubicin, and cyclophosphamide alternating with ifosfamide and etoposide (VDC/IE) was initiated before a definitive diagnosis was established. MRI demonstrated gradual tumor shrinkage during treatment (**Figures 2C–E**). After five cycles of VDC/IE chemotherapy, the patient underwent CIRT with a total dose of 70.4 Gy delivered in 16 fractions over 4 weeks, as previously reported (5–7). CIRT was planned using a CT-based three-dimensional treatment planning system. The patient was immobilized in the prone position using a custom-made thermoplastic cast. Target volumes and organs at risk were delineated on CT-MRI fusion images. Carbon-ion beams were delivered by passive scattering, and treatment was performed on 4 consecutive days per week from Tuesday to Friday (**Figure 3**), followed by 12 cycles of adjuvant chemotherapy consisting of VDC, VC, and IE regimens, with radiological assessments showing SD, followed by PR, and ultimately CR at week 50 (**Figure 3D**). No obvious residual mass was detected on MRI at the completion of chemotherapy (**Figure 4A**), and there was no evidence of distant metastasis. After the concept of BCOR-rearranged sarcoma became widely recognized, additional genetic testing using RNA extracted from frozen biopsy specimens. Subsequent RT-PCR was conducted using previously reported primers described by Perron et al., followed by Sanger sequencing, which confirmed the presence of the *BCOR–CCNB3* fusion gene (**Figure 4B**), leading to a final diagnosis of BCS.

**Figure 2:**
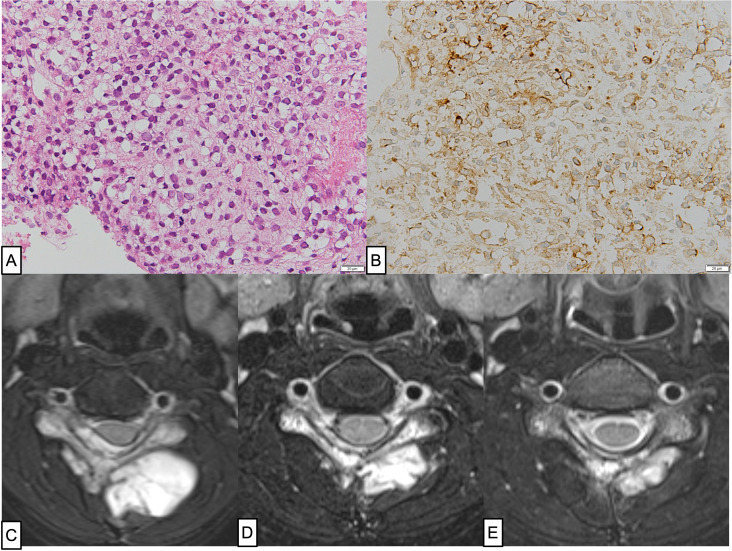
Histopathological and radiological findings during neoadjuvant chemotherapy. Needle biopsy specimen showing an undifferentiated round-cell sarcoma **(A)**. Immunohistochemical staining demonstrates that the tumor cells are positive for CD99 **(B)**. Serial magnetic resonance imaging (MRI) obtained during neoadjuvant chemotherapy shows a gradual reduction in tumor size at 1 month **(C)**, 2 months **(D)**, and 3 months **(E)** after treatment initiation.

**Figure 3:**
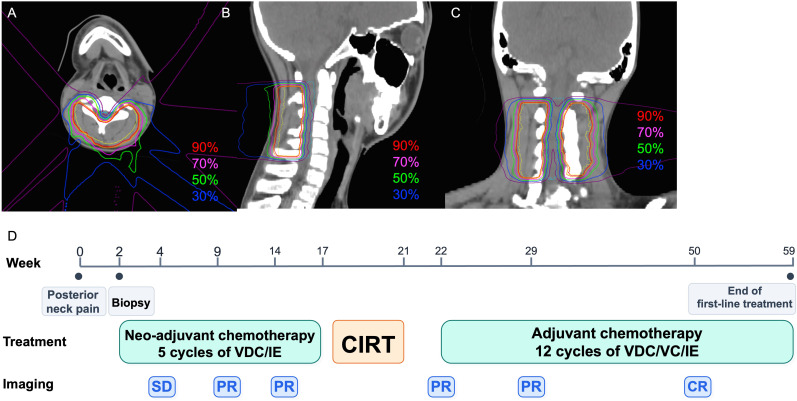
Dose distribution of carbon-ion radiotherapy, and clinical course of the patient. Axial **(A)**, sagittal **(B)**, and coronal **(C)** images illustrating the radiotherapy dose distribution. The image show the dose distribution within the tumor. Ninety percent of the total dose is shown in red, 70% in pink, 50% in green, and 30% in blue. Timeline demonstrate the patient’s clinical course from symptom onset through biopsy, neoadjuvant chemotherapy (5 cycles of VDC/IE), CIRT, and adjuvant chemotherapy (12 cycles of VDC/VC/IE), with radiological assessments demonstrating SD, followed by PR, and ultimately CR at week 50 **(D)**. CIRT, carbon-ion radiotherapy; VDC, vincristine/doxorubicin/cyclophosphamide; IE, ifosfamide/etoposide; VC, vincristine/cyclophosphamide; SD, stable disease; PR, partial response;CR, complete response.

**Figure 4:**
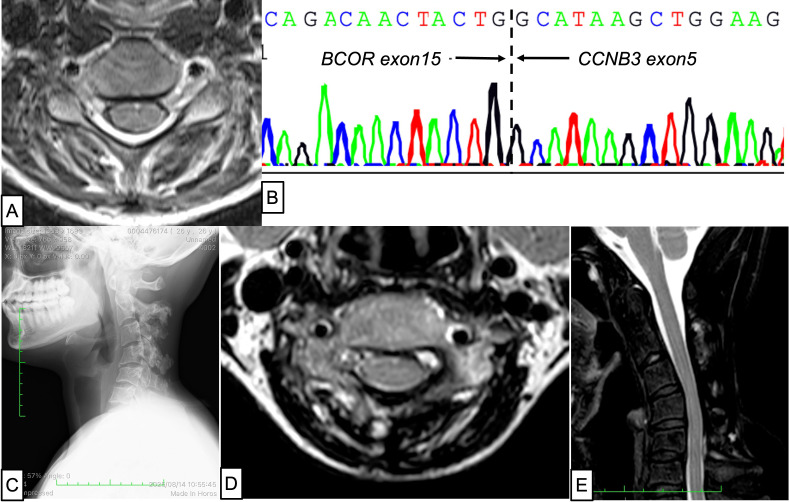
Post-treatment imaging and molecular findings. Magnetic resonance imaging (MRI) performed after treatment completion showed no evidence of residual tumor **(A)**. Sanger sequencing confirms the presence of the BCOR–CCNB3 fusion gene **(B)**. At the 12-year follow-up, radiography demonstrated kyphosis **(C)**; however, there was no evidence of tumor recurrence or spinal cord compression on follow-up imaging **(D, E)**.

More than 12 years after treatment, follow-up radiographs demonstrated cervical kyphosis (**Figure 4C**). However, the patient remained completely asymptomatic without neurological deficits, and there was no evidence of local recurrence (**Figures 4D, E**) or distant metastasis.

## 3 Discussion

Here, we present a rare case of BCS arising in the cervical spine. Historically, many tumors that are now recognized as BCS were diagnosed as Ewing-like sarcomas. Consequently, treatment strategies have frequently followed those used for ES, including perioperative chemotherapy and local treatments, such as surgery and/or radiotherapy, although the optimal regimen remains controversial (3). The treatment outcomes of BCS have been reported to be comparable to those of ES, with reported 5-year survival rates of approximately 75% (3). However, owing to its rarity, the 5-year survival rate of patients with BCS involving the spine has not been well documented. Based on the available data for ES arising outside the appendicular skeleton, the 5-year survival rate is estimated to be 40%–60% (8).

In the present case, the tumor was initially diagnosed as an Ewing-like sarcoma because its histological features resembled those of ES in the absence of *EWSR1* rearrangement. Considering the concern regarding rapid tumor progression with spinal cord compression, systemic chemotherapy based on an ES protocol (VDC/IE) was initiated. Subsequent genetic analysis confirmed the presence of the *BCOR–CCNB3* fusion gene, which led to a definitive diagnosis of BCS.

Owing to the rarity of spinal BCS, an optimal treatment strategy has yet to be established. Although wide surgical resection is generally preferred as a local treatment for BCS (3), the management of spinal sarcomas is challenging because complete surgical resection with adequate margins is often difficult because of the proximity of critical neurological structures (4, 5). In the present patient, neoadjuvant chemotherapy resulted in significant tumor shrinkage, including regression of intraspinal extension. Recent reports have suggested that the extent of surgical resection in Ewing sarcoma may be determined based on such residual tumor following neoadjuvant chemotherapy (9), and based on this concept, complete resection with adequate margins might have been considered feasible in the present case. However, this concept had not yet been established at the time of treatment, and pretreatment MRI findings were used to define the intended surgical margins, as remains the most common approach today (10). In the present case, pretreatment MRI demonstrated that the tumor arose from the posterior elements of C3 with intraosseous spread within the posterior arch, intraspinal extension, and close proximity to the vertebral artery. These anatomical characteristics imposed critical constraints on the achievable surgical margins. Specifically, obtaining adequate oncological margins for the intraosseous component would have necessitated resection extending to the vertebral artery, carrying a substantial risk of vascular injury. Furthermore, achieving clear margins for the intraspinal component would have required resection of the dura mater, with attendant risks of cerebrospinal fluid leakage, meningitis, and significant neurological morbidity including spinal cord injury. These findings are consistent with previously reported cases in which adequate surgical margins could not be achieved due to comparable anatomical constraints (11, 12). Given these considerations, wide resection with adequate oncological margins was deemed technically infeasible and associated with unacceptable risks of residual disease and permanent neurological deficits. In such situations, radiotherapy, including particle-beam therapy, is often employed in the management of ES and has been associated with favorable local control (4, 5).

CIRT has emerged as a promising treatment modality for unresectable bone and soft tissue sarcomas (6, 13). Sarcomas are considered relatively radioresistant tumors; therefore, higher total doses and higher fractional doses are necessary compared with conventional photon radiotherapy. Compared with conventional photon radiotherapy, carbon ions provide superior dose distribution through the Bragg peak and exhibit higher relative biological effectiveness owing to their high linear energy transfer, enabling greater dose concentration within the tumor while minimizing irradiation of adjacent normal tissues such as the spinal cord — making a hypofractionated regimen both physically rational and clinically feasible (13). These characteristics are particularly advantageous for tumors located in anatomically complex regions, such as the spine (14), and may be especially beneficial in pediatric patients by reducing the integral dose to normal tissues. CIRT for sarcoma has been performed since 1996 at a single institute in Japan, with the total dose determined based on previous clinical trials by Kamada et al. and Imai et al. (7, 13). Accordingly, a hypofractionated regimen consisting of 16 fractions with a higher dose per fraction was adopted.

Iwata et al. reported that a combination of high-dose chemotherapy and CIRT for ES achieved good local control but failed to prevent distant metastases (5). By contrast, the present patient demonstrated a favorable clinical course with no evidence of local recurrence or distant metastasis for more than 12 years. These long-term outcomes suggest that multimodal therapy combining chemotherapy and CIRT may provide durable disease control in patients with spinal BCS. Furthermore, Merjaneh et al. reported that a patient with sarcoma harboring a *BCOR* gene alteration who received VDC/IE chemotherapy and proton radiotherapy survived with no evidence of disease at 40 months (15).

Photon stereotactic body radiation therapy (SBRT) represents another modern local treatment option for selected spinal sarcomas and has produced encouraging local control rates in retrospective series; however, these cohorts are predominantly composed of metastatic cases, include mixed sarcoma histologies without Ewing sarcoma-specific outcome data, and remain relatively small in size (16). In contrast, CIRT has demonstrated durable local control in unresectable primary spinal sarcoma (6, 13), supporting its consideration when maximal local intensification is required at an anatomically constrained site such as the cervical spine. Taken together with the present case, particle-beam therapy may be a promising treatment option for sarcomas with BCOR alterations, particularly for unresectable cases.

However, a complete response to chemotherapy has also been reported in patients with BCS (3). Therefore, it remains difficult to determine whether the favorable outcome in the present patient was attributable to chemotherapy or CIRT. In addition, the long-term complications of CIRT in pediatric patients remain largely unknown; therefore, careful long-term follow-up is warranted, and further accumulation of cases is necessary to clarify the optimal management strategy for spinal BCS.

In conclusion, we report a rare case of BCS arising from the cervical spine with a long-term follow-up. This case suggests that multimodal treatment including chemotherapy and CIRT may achieve durable disease control when complete surgical resection is difficult. Further accumulation of cases is required to clarify the optimal management of spinal BCS.

## Data Availability

The raw data supporting the conclusions of this article will be made available by the authors, without undue reservation.

## References

[1] Pierron G Tirode F Lucchesi C Reynaud S Ballet S Cohen-Gogo S . A new subtype of bone sarcoma defined by BCOR-CCNB3 gene fusion. Nat Genet. (2012) 44:461–6. doi: 10.1038/ng.1107 22387997

[2] Board W World Health Organization International Agency for Research on Cancer . Soft Tissue and Bone Tumours. Geneva. (2020). pp. x–607.

[3] Imanishi J Sato K Kikuchi Y Yamamoto A Watabe S Matsuyama T . Update on the management of BCOR::CCNB3 sarcoma. Jpn J Clin Oncol. (2025) 55:1097–104. doi: 10.1093/jjco/hyaf111 40652565

[4] Indelicato DJ Vega RBM Viviers E Morris CG Bradfield SM Ranalli NJ . Modern therapy for spinal and paraspinal Ewing sarcoma: An update of the University of Florida experience. Int J Radiat Oncol Biol Phys. (2022) 113:161–5. doi: 10.1016/j.ijrobp.2022.01.007 35033584

[5] Iwata S Yonemoto T Ishii T Kumagai K Imai R Hagiwara Y . Efficacy of carbon-ion radiotherapy and high-dose chemotherapy for patients with unresectable Ewing's sarcoma family of tumors. Int J Clin Oncol. (2013) 18:1114–8. doi: 10.1007/s10147-012-0480-y 23053401

[6] Imai R Kamada T Araki N . Carbon ion radiation therapy for unresectable sacral chordoma: An analysis of 188 cases. Int J Radiat Oncol Biol Phys. (2016) 95:322–7. doi: 10.1016/j.ijrobp.2016.02.012 27084649

[7] Imai R Kamada T Tsuji H Sugawara S Serizawa I Tsujii H . Effect of carbon ion radiotherapy for sacral chordoma: Results of Phase I-II and Phase II clinical trials. Int J Radiat Oncol Biol Phys. (2010) 77:1470–6. doi: 10.1016/j.ijrobp.2009.06.048 19939576

[8] Ansari MN Gupta A Jain DK Rana D . The Ewing's sarcoma of cervical spine-a rare occurrence. Spinal Cord Ser cases. (2022) 8:68. doi: 10.1038/s41394-022-00534-6 35864101 PMC9304365

[9] Thévenin-Lemoine C Destombes L Vial J Wargny M Bonnevialle P Lefevre Y . Planning for bone excision in Ewing sarcoma: Post-chemotherapy MRI more accurate than pre-chemotherapy MRI assessment. J Bone Joint Surg Am. (2018) 100:13–20. doi: 10.2106/JBJS.16.01461 29298256

[10] Nogaro MC Mifsud M McCarthy C Orosz Z Pratap S Craig R . The safe surgical margin in Ewing's sarcoma. Surg Oncol. (2022) 41:101737. doi: 10.1016/j.suronc.2022.101737 35358914

[11] Khatavi A Dhillon CS Chhasatia N Pophale C Nanakkal S Varshney A . Primary Ewing's sarcoma of the C2 vertebra with progressive quadriparesis: Report of a rare case and review of the literature. Surg Neurol Int. (2020) 11:340. doi: 10.25259/SNI_497_2020 33194274 PMC7656003

[12] Sewell MD Tan KA Quraishi NA Preda C Varga PP Williams R . Systematic review of en bloc resection in the management of Ewing's sarcoma of the mobile spine with respect to local control and disease-free survival. Med (Baltimore). (2015) 94:e1019. doi: 10.1097/MD.0000000000001019 26166072 PMC4504586

[13] Kamada T Tsujii H Tsuji H Yanagi T Mizoe JE Miyamoto T . Efficacy and safety of carbon ion radiotherapy in bone and soft tissue sarcomas. J Clin Oncol. (2002) 20:4466–71. doi: 10.1200/JCO.2002.10.050 12431970

[14] Matsumoto K Imai R Kamada T Maruyama K Tsuji H Tsujii H . Impact of carbon ion radiotherapy for primary spinal sarcoma. Cancer. (2013) 119:3496–503. doi: 10.1002/cncr.28177 23939877

[15] Merjaneh N Kim H Escoto H Metts J Ray A Bukowinski A . Strategies for the treatment of infantile soft tissue sarcomas with BCOR alterations. J Pediatr Hematol Oncol. (2023) 45:315–21. doi: 10.1097/MPH.0000000000002620 36706311 PMC11225610

[16] Kim E Kim MS Paik EK Chang UK Kong CB . Treatment outcomes of stereotactic body radiation therapy for primary and metastatic sarcoma of the spine. Radiat Oncol. (2023) 18:156. doi: 10.1186/s13014-023-02346-w 37736735 PMC10514933

